# Hypertrophic Cardiomyopathy: Updates Through the Lens of Sports Cardiology

**DOI:** 10.1007/s11936-021-00934-1

**Published:** 2021-05-25

**Authors:** Bradley S. Lander, Dermot M. Phelan, Matthew W. Martinez, Elizabeth H. Dineen

**Affiliations:** 1 Division of Cardiology, Columbia University Irving Medical Center, New York, NY 10032 USA; 2 Sanger Heart & Vascular Institute, Atrium Health, Charlotte, NC 28203 USA; 3 Department of Cardiovascular Medicine, Atlantic Health, Morristown Medical Center, Morristown, NJ 07960 USA; 4 Sports Cardiology and Hypertrophic Cardiomyopathy, 111 S Madison Ave, Suite 300, Morristown, NJ 07960 USA; 5 Division of Cardiology, University of California Irvine, 333 City Blvd W, Suite 400, Orange, CA 92868 USA

**Keywords:** Hypertrophic cardiomyopathy, Sports cardiology, Athlete, Exercise

## Abstract

**Purpose of review:**

This review will summarize the distinction between hypertrophic cardiomyopathy (HCM) and exercise-induced cardiac remodeling (EICR), describe treatments of particular relevance to athletes with HCM, and highlight the evolution of recommendations for exercise and competitive sport participation relevant to individuals with HCM.

**Recent findings:**

Whereas prior guidelines have excluded individuals with HCM from more than mild-intensity exercise, recent data show that moderate-intensity exercise improves functional capacity and indices of cardiac function and continuation of competitive sports may not be associated with worse outcomes. Moreover, recent studies of athletes with implantable cardioverter defibrillators (ICDs) demonstrated a safer profile than previously understood. In this context, the updated American Heart Association/American College of Cardiology (AHA/ACC) and European Society of Cardiology (ESC) HCM guidelines have increased focus on shared decision-making and liberalized restrictions on exercise and sport participation among individuals with HCM.

**Summary:**

New data demonstrating the safety of exercise in individuals with HCM and in athletes with ICDs, in addition to a focus on shared decision-making, have led to the most updated guidelines easing restrictions on exercise and competitive athletics in this population. Further athlete-specific studies of HCM, especially in the context of emerging therapies such as mavacamten, are important to inform accurate risk stratification and eligibility recommendations.

## Introduction

Hypertrophic cardiomyopathy (HCM) is a heterogenous and common inherited cardiac disorder defined by maximal left ventricular (LV) wall thickness ≥ 15 mm and disproportionate to loading conditions [1, 2••]. The diagnosis can also be made with lesser degrees of LV hypertrophy (LVH) in individuals with a positive family history or known genetic mutations [1, 2••]. The combined prevalence of clinically expressed HCM and gene carriers has been recently estimated at 1:200 [3], though it is likely lower in highly trained athletes as structural and functional changes associated with the disorder select out many individuals from competitive athletics [4]. Historically, sports activity has been associated with increased risk of sudden cardiac arrest (SCA) among individuals with underlying cardiovascular disease [5] and HCM was thought to be the most common cause of death in young athletes [6, 7]. As a result, prior guidelines restricted individuals with clinically apparent HCM from most competitive sports [1, 8–11]. However, this paradigm has evolved. This review will highlight updated guidelines, with relevance to sports cardiology, for the diagnosis, risk stratification, treatment, and exercise recommendations for individuals with HCM.

## Diagnosis

HCM is an autosomal-dominant condition caused by variants in sarcomeric protein genes defined by maximal end-diastolic LV wall thickness ≥ 15 mm in one or more LV myocardial segments as measured by echocardiography, cardiac magnetic resonance imaging (CMR), or computed tomography (CT), and disproportionate to hemodynamics or other pathological conditions such as long-standing hypertension or severe aortic stenosis [1, 2••]. The diagnosis can also be made in individuals with lesser degrees of hypertrophy who have a positive family history or genetic mutations [1, 2••]. Surveillance imaging is critical to monitor for the development or worsening of high-risk features and is particularly important for individuals who are genotype-positive but at first evaluation phenotype-negative.

### Gray zone

Intense physical training is associated with cardiac remodeling. The details of one’s remodeling depend on numerous factors including age, race, gender, and the type of sport and intensity [12–14]. Classically, power/static activities (such as weightlifting, American football, or shotput) are associated with increased afterload and peripheral vascular resistance with less pronounced effects on cardiac output. As such, they are characterized more by LVH and less chamber dilation. On the contrary, the consistent volume challenge associated with endurance sports (such as running) is more often associated with chamber dilation and augmented cardiac output [15]. However, the greatest degree of LVH occurs in those who also have the greatest increase in LV cavity size, the mixed sport athlete [14]. Approximately 2% of white athletes and up to 18% of black athletes have LVH with wall thickness in the “gray zone”; that is, less than the diagnostic threshold for HCM but above usual measurements, 13–14 mm in men and 12–13 mm in women [15–22]. Differentiating HCM from exercise-induced cardiac remodeling (EICR) is challenging but has important therapeutic implications.

### Differentiating EICR and HCM

#### Electrocardiogram (ECG)

Although 5–10% of individuals with HCM have normal ECGs [23, 24], the majority demonstrate abnormalities including deep T-wave inversions (TWI), particularly in the inferolateral leads, ST segment depression or elevation, pathologic Q waves, and intraventricular conduction delay, which are not expected from athletic training [23, 25]. When TWI are isolated to leads aVR, III, or V1, they may be considered a normal variant and in Black athletes may be considered normal when TWI in V1–V4 are preceded by domed ST segments [26]. However, in one study, approximately 5% of Black male athletes and 1.6% of Black female athletes demonstrated pathologic TWI and nearly half of all athletes presenting with pathologic TWI were found to have identifiable cardiac disease, most commonly HCM [27]. The International Recommendations for Electrocardiographic Interpretation in Athletes were published in 2017 and serve as a valuable resource for clinicians who review the ECGs of athletes [25]. Isolated QRS voltage criteria for LVH are commonly seen in athletes; however, further evaluation is required when accompanied by two or more borderline ECG features such as atrial enlargement and axis deviation [25]. One study found that a Q + S wave amplitude > 1.0 mV in lead III was associated with septal thickness and more likely in individuals with HCM when compared with athletes; however, this study is not included in the ECG interpretation guidelines [28]. Abnormal ECG findings should prompt further investigation with imaging.

#### Echocardiography

Transthoracic echocardiography (TTE) is routinely used to evaluate suspected structural abnormalities. As opposed to athletes in which LVH is commonly symmetric and associated with LV chamber dilation, in HCM, the most common location for LVH is the basal anterior septum in continuity with the anterior free wall, though focal hypertrophy in only one or two LV segments is possible [2••]. Other features favoring HCM include myocardial crypts and abnormalities of the structure and function of the mitral valve and subvalvular apparatus including hypertrophied and apically displaced papillary muscles, anomalous insertion of the papillary muscle directly onto the anterior leaflet of the mitral valve, accessory chordal attachments, elongated mitral valve leaflets, and systolic anterior motion (SAM) of the mitral valve [2••].

Several studies demonstrate that even in highly trained white athletes, LVH > 13 mm is extremely rare and, as such, measurements outside of this range should be investigated further [4, 16, 29, 30]. However, the extent of LVH is greater in men, and in older, larger and Black athletes [13, 14, 31, 32].

In “gray zone” cases, athletes tend to have larger LV cavities than patients with HCM and an LV cavity size < 54 mm distinguished sedentary patients with HCM from athletes with physiologic remodeling in one small study [19] and < 51 mm distinguished between athletes with HCM and physiologic remodeling in another study [23]. Moreover, diastolic function is most often normal in athletes with LVH as compared to patients with HCM [19, 33, 34]. The caveat is that studies of diastolic function were not focused on strength-trained athletes, those most likely to have concentric LVH, and a recent study of American-style football players with concentric LVH demonstrated relative impairment of LV diastolic function which may be explained by resting hypertension in the population [35, 36]. Another study showed that athletes with HCM, as opposed to sedentary HCM patients, can have normal indices of diastolic function, thus diminishing its ability to distinguish certain cases in the “gray zone” [23].

Because up to one-third of individuals with HCM have latent left ventricular outflow tract (LVOT) obstruction, dynamic maneuvers, including exercise stress echocardiography, should be used to detect an exercise-induced LVOT gradient especially in the presence of exercise-induced symptoms [37].

#### Cardiac magnetic resonance imaging

Cardiac magnetic resonance (CMR) is an excellent diagnostic modality for proper assessment of chamber size, mass, and visualizing areas which may not be optimally visualized by TTE including the anterior free wall, posterior septum, and apex [2••]. CMR can identify late gadolinium enhancement (LGE) suggestive of fibrosis, which can be seen in 75% of people with HCM, most often in the segment of maximal wall thickness and at the anteroseptal and inferoseptal segments of insertion of the right ventricle [38]. Whereas LGE may be seen in long-term endurance athletes [39–41], its presence in young athletes has been less well described. LGE patterns suggestive of prior infarction or healed myocarditis are associated with increased cardiovascular risk, but less is known about the significance of a non-specific LGE pattern at the RV insertion points especially in asymptomatic individuals. Data on patterns of LGE among COVID-19 patients and comparable controls is still emerging and requires ongoing research. In one study of athletes with pathological TWI, initial TTE missed 46% of all diagnosed pathologic cases and CMR provided a diagnosis in 88% of all cardiomyopathies including 30% with “suspicious TTEs” [27]. Most of these were apical HCM which can be missed by TTE due to the limitations of echocardiographic image acquisition.

#### Cardiopulmonary exercise testing

Cardiopulmonary exercise testing (CPET) may be marginally helpful to differentiate HCM from EICR [2••]. One study compared athletic men with genetically proven HCM and mild LVH to elite athletes matched for age, size, and LV wall thickness and showed a greater peak oxygen uptake (pVO2), anaerobic threshold, and oxygen pulse among elite athletes [42]. A pVO2 > 50 ml/kg/min or > 20% above the predicted VO2 max, anaerobic threshold > 55% of predicted VO2 max, and an oxygen pulse > 30 ml/beat differentiated athlete’s heart from HCM [42]. Notably, the subjects with HCM were not elite athletes, and in our experience, some athletes with HCM can achieve metrics comparable to the elite athletes in this study.

#### Detraining

Detraining involves stopping rigorous exercise to evaluate the effects on cardiac structure and function. It is used in certain cases in which the gray zone of hypertrophy remains undifferentiated despite a complete workup. Although there have been no studies in athletes with HCM detailing regression of hypertrophy, several small studies of athletes with eccentric hypertrophy [43, 44] and one small study of athletes with concentric hypertrophy have documented regression following periods of detraining [21]. This strategy should always be utilized with caution due to lack of normative data for the rate and completeness of expected LVH regression in healthy athletes, though atrophy of LV mass at a rate of ~1%/week has been observed during strict bedrest in a non-athlete cohort [45]. This strategy may be challenging for elite athletes whose job or livelihood is dependent on maintaining peak physical performance.

## Risk stratification

Despite a normal life expectancy for most individuals with HCM [46] and data suggesting that most deaths are unrelated to HCM [47], the disease has been considered the most common and visible cause of SCA among young athletes in North America. This underscores the importance of distinguishing HCM from EICR [2••, 6, 7, 48, 49]. Risk stratification, especially of individual athletes, is challenging. The mechanism of death is thought to be from ventricular arrhythmias in the setting of myocardial disarray, interstitial collagen deposition, and scarring after myocyte death from microvascular dysfunction and ischemia, thus creating an unpredictable arrhythmogenic substrate [49]. A recent meta-analysis, however, suggested that a structurally normal heart was more common than HCM in young individuals with SCA [50]. This study suggested that, in total, there was no significant difference between HCM and structurally normal hearts among athletes, except for in Europe where structurally normal hearts were more common [50]. Repeated risk assessment for SCA every 1–2 years is critical for patients with HCM in order to identify high-risk patients who may benefit from an implantable cardioverter defibrillator (ICD).

### History

A detailed personal and family history is paramount to risk stratification. Risk factors to consider include younger age at the time of evaluation, personal history of aborted cardiac arrest, unexplained syncope (particularly within 6 months as those occurring > 5 years in the past have less significance), or a family history of sudden death, SCA, or sustained ventricular arrhythmias (VA) in ≥ 1 first-degree relative ≤ 50 years old or SCA in a first-degree relative with confirmed HCM (pre or post death diagnosis) at any age [1, 2, 51, 52, 53••]. Hypertension and epicardial coronary disease may also portend a worse prognosis in patients with HCM [54, 55]. Moreover, as Drezner et al. recently discuss, Black athletes and male basketball, soccer, and American football players all appear to be at increased risk for SCA [56].

### Rhythm monitoring

While the resting ECG has limited value, 24–48-h Holter or continuous 30-day ambulatory monitoring is more sensitive to detect non-sustained ventricular tachycardia (NSVT) and sustained VA and should include an exercise session [1, 2••, 51, 52, 53••, 54]. Patients with HCM with prior SCA or sustained VA have the highest risk of subsequent events, about 10% per year [11].

### Echocardiography

Echocardiography is used to evaluate maximal wall thickness in the parasternal short and long axis planes, LVOT gradient (at rest and with Valsalva), and left atrial (LA) diameter. Exercise stress echocardiography can be used in individuals with exertional symptoms and resting SAM but absent or mild LVOT obstruction [1, 2••, 51, 52, 53••]. Echocardiographic features which portend a worse prognosis include an LV ejection fraction < 50%, apical aneurysm, and wall thickness > 30 mm [11, 49, 57].

### Cardiac magnetic resonance imaging

Though LGE may be present in up to 75% of HCM patients, involvement of ≥ 15% of LV myocardium can identify individuals at higher risk of SCA and VA, even in the absence of other conventional risk factors [58]. Absence of LGE is associated with a lower risk of SCA and is more reassuring [58]. Furthermore, CMR can provide further characterization of maximal LV wall thickness, ejection fraction, and the presence of an LV apical aneurysm [2••, 58–62].

### Exercising testing

Exercise testing should be a part of routine evaluation. Abnormal blood pressure (BP) response (defined as < 20 mmHg increase in systolic BP from baseline or exercise-induced hypotension) was thought to portend a worse prognosis but the evolution of SCA risk assessment has led to its removal as a routine marker of increased SCA risk [2••]. Exercise-induced symptoms should lead to more conservative exercise/sporting participation recommendations [53••]. Exercise testing with echocardiographic guidance can aid in risk stratification. Maximal left ventricular outflow tract (LVOT) gradient to aid in calculating an estimated 5-year sudden death risk may be useful during shared decision-making for implantable ICD placement [2••].

### Genetic testing

Genetic testing should be performed for familial screening, but because of significant genetic heterogeneity, does not inform risk of SCA or contribute to exercise recommendations [53••, 57].

### Risk prediction models

The ESC published a model to estimate individual 5-year risk of SCA to provide guidance on the need for prophylactic ICDs. The model incorporates seven variables: age, syncope, family history of SCA from HCM, maximal LV wall thickness, LA diameter, LVOT obstruction, and NSVT [1, 51]. The guidelines delineate low (< 4%), moderate (4–6%), and high risk (≥ 6%). Notably, this was not created for athletes specifically and may not represent the true risk of SCA for athletes with HCM. It also does not account for LGE, reduced systolic function, or an LV apical aneurysm. Other studies have found the ESC-HCM model to have a low sensitivity for SCA and argue that the calculator, based on mathematical population models, leaves many individuals vulnerable [57, 63, 64]. Specifically, Maron et al. followed 2094 patients with HCM over 17 years and showed that the ESC risk score was less sensitive compared with an enhanced ACC/AHA risk factor strategy [65••]. Using this strategy, one of the following seven risk factors was considered sufficient evidence of increased SCA risk to justify prophylactic ICD implantation: (1) family history of SCA definitively or likely caused by HCM in ≥ 1 first-degree or other close relative ≤ 50 years old, (2) LVH with wall thickness > 30 mm, (3) unexplained syncope unlikely to be vasovagal within 5 years of evaluation, (4) NSVT, (5) extensive LGE (> 15% LV mass), (6) LV ejection fraction ≤ 50%, and (7) LV apical aneurysm [65••]. A recent review estimated the annual risk of SCA in young athletes with HCM to be between 0.1 and 6.6% per year [56]. Importantly, these data are extrapolated from small numbers in three studies of athletes who may not have had an established diagnosis of HCM and therefore potentially harbor significant risk factors. The risk of participation among low-risk athletes actively followed in sports cardiology clinics is likely lower. Maron et al. have demonstrated that SCA risk stratification has improved over time and the risk of SCA in those deemed “low risk” is extremely low [65••, 66].

## Treatment

For patients with HCM, limitations to exercise tolerance are multifactorial and include LVOT obstruction [67]; diastolic dysfunction [68, 69]; abnormal hemodynamic response to exercise [70, 71]; predisposition to arrhythmias, including atrial fibrillation and NSVT [72–74]; mitral valve abnormalities [75]; and myocardial ischemia [76, 77]. Multimodal treatments in athletes include lifestyle adjustments, pharmacotherapy, and invasive options, and are primarily aimed at relief of symptoms associated with LVOT obstruction and prevention of SCA.

### Lifestyle

Proper hydration augments preload and is important for the two-thirds of HCM patients with obstruction; therefore, it is prudent to avoid dehydrating situations such as hot tubs, hot showers, and saunas, as well as exercising during concomitant illness. It is also recommended to avoid stimulants and the acute changes in preload and afterload that come with start-stop activities. However, regular moderate-intensity exercise is beneficial and should be encouraged in most patients with HCM as will be discussed later. For individuals with HCM who plan to participate in high-intensity activities after shared decision-making with an expert in the field, an emergency action plan is important and should include access to an automated external defibrillator (AED).

### Pharmacotherapy

The most common and relevant issues that active individuals with HCM must manage include LVOT obstruction and associated symptoms, treated predominantly by negative inotropes, as well as the risk of SCA, addressed through the potential implantation of an ICD. Individuals with obstructive HCM should avoid pure vasodilators such as dihydropyridine calcium channel blockers, angiotensin-converting enzyme inhibitors, and angiotensin receptor blockers which decrease afterload and promote LVOT obstruction. Similarly, while low-dose diuretics may be necessary for individuals with congestive symptoms and those with non-obstructive HCM, high-dose diuretics, which may cause preload insufficiency, should be used cautiously and sparingly. [2••]

Beta blockade: First-line therapy includes non-vasodilating beta blockers titrated to a heart rate of approximately 60 beats per minute or relief of symptoms. Its efficacy is a result of negative inotropy, negative chronotropy, reduced LVOT gradients, increased systolic ejection time, and improved LV filling.

Considerations in active individuals: May produce unintended side effects such as fatigue, lethargy, or decreased cardiorespiratory fitness [78]. Use may be limited by baseline bradycardia in well-conditioned individuals. In general, they are well tolerated by both active and non-active individuals.

Non-dihydropyridine calcium channel antagonists: If beta blockers are ineffective, non-dihydropyridine calcium channel blockers, such as verapamil or diltiazem, can be used. These medications are negative inotropes and chronotropes and prolong diastolic filling time.

Considerations in active individuals: Caution in active individuals with baseline low blood pressure given the potential for vasodilation and hypotension. Peripheral edema is rare but may be more bothersome to this population than to sedentary individuals.

Disopyramide: Although less commonly used in active individuals, this is an option in individuals with incomplete response to beta blockers or calcium channel blockers and its benefit comes from strong negative inotropy.

Considerations in active individuals: Its anticholinergic properties, such as dry mouth and blurry vision, could affect performance. The need for hospital admission to monitor for potential arrhythmogenicity may disrupt training or competition schedules. [79] As a result of this potential side effect, guidelines recommend concomitant use of an atrioventricular nodal-blocking agent [2••].

### Septal reduction therapy

Septal reduction therapy with surgical myectomy is rarely considered in athletes with the possible exception of refractory exertional syncope in athletes with dynamic LVOT obstruction not attributable to other causes. Such cases are rare and not described in the guidelines.

### Implantable cardioverter defibrillators

Athletes may receive ICDs for secondary prevention following a life-threatening VA or for primary prevention according to disease-specific risk stratification. Based on limited data, prior consensus recommendations restricted athletes with ICDs to low-moderate dynamic and low static (1A) sports such as bowling or golf [80–83]. The concerns that led to these recommendations included the unknown efficacy of ICDs during exercise, the possibility of device malfunction, risk of injury to the athlete, or device from trauma and the potential for appropriate or inappropriate discharges.

The ICD Sports Registry was developed to follow athletes with ICDs who continued to compete in sports of greater intensity than the “1A” recommendation. Though shocks were common in 44 months of follow-up, there were no ICD failures, external resuscitations, injuries due to syncopal arrhythmias, or loss of control after shock [84]. More shocks were received during physical activity than rest, but there was no difference between competition or practice and other physical activity [84]. Importantly, though this registry includes athletes across numerous sports and cardiac conditions, no specific sub-analysis has been done of HCM patients. As a result of data from the ICD Sports Registry, the AHA/ACC updated guidelines note that participation in sports with an ICD is now a “IIb” recommendation, indicating it “may be considered”. [85]

A sub-analysis of this registry performed to address appropriate ICD programming showed that inappropriate shocks were less likely with higher rate cut-offs for the first therapy zone (greater than 200 beats per minute) and with detection duration greater than “nominal” settings [86]. Individuals with both of those programming features were the least likely to receive inappropriate shocks. Importantly, there was no increase in syncope prior to shock in the athletes with these programming features. The presence of single or dual chamber devices and the number of therapy zones were not related to risk of shock Exercise testing after implantation may be helpful to determine the maximum sinus rate and to avoid T-wave oversensing [86].

Participants in the ICD Sports Registry had transvenous devices (except for a few epicardial systems). It is unknown whether subcutaneous-ICDs (S-ICD) are as effective or protected from lead damage to the same degree in athletes. In a recent review, Lampert described that athletes in sports requiring intense arm use, such as swimmers or rowers, may favor S-ICD due to the lower likelihood of “subclavian crush syndrome” in which the transvenous lead becomes compressed between the clavicle and first rib [87]. The review also noted that the position of the generator may drive the decision in specific contact sports; for example, the pre-pectoral placement of the generator in transvenous devices is better protected by standard padding in American football or ice hockey than an S-ICD which is located along the lateral, lower rib cage [87].

As was the case in prior guidelines, the most recent American HCM guidelines emphasize that ICDs should not be placed solely to allow for sports participation and do not serve as a substitute for disease-related recommendations that restrict participation [2••].

## Exercise and competitive sport recommendations

### Why is there concern about exercise and HCM?

**Fig. 1:**
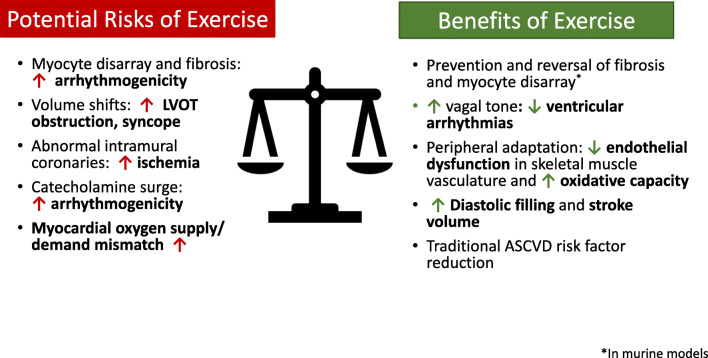
Exercise in hypertrophic cardiomyopathy.

While regular physical activity is effective for primary and secondary prevention of numerous chronic diseases [88], there is concern that exercise and high-intensity sports may cause maladaptive remodeling, VA and SCA among individuals with HCM [89]. The concern over risk is based on several proposed mechanisms (Fig. 1) [89–91]. Prior studies offer conflicting data regarding the true risk of SCA during exercise and competition and the proportion of which are due to HCM, with more recent studies in the general population and among athletes suggesting that structurally normal hearts are more common in SCA than previously thought [5–7, 48, 50, 92, 93]. Moreover, recent studies have shown that two-thirds of SCA in younger individuals with HCM died during routine activities, rest, or sleep [94], and was corroborated by a general population-based study in children and young adults [95]. Importantly, although a smaller proportion died during activity, the amount of time spent during the day in activity is less than the time at rest. However, a recent analysis by Maron et al. showed that 36% of SCA in young competitive athletes with an autopsy-confirmed cardiovascular diagnosis were due to HCM and emphasizes the danger of trivializing the HCM-associated risk [96]. Based on similar data, guidelines historically have limited individuals with HCM to low-intensity, “1A” sports [8, 9, 82, 97]. Perhaps as a result of messaging regarding the risks of exercise, surveys of individuals with HCM report that they are less active, more obese, and negatively affected emotionally compared to normal controls [98–100].

### What research has been done on HCM and exercise?

Research in murine models with a mutant myosin heavy chain has shown a protective effect of early exercise on fibrosis and myofibrillar disarray when compared to exercise started later in life, suggesting that exercise may be beneficial especially early in the disease course. [101]

In humans, several studies have demonstrated the benefits of regular, moderate exercise in patients with HCM. The RESET-HCM trial showed that a 16-week, moderate-intensity exercise program modestly increased peak VO2 compared with placebo [102] and Klempfner et al. showed that a supervised, gradual exercise program in symptomatic HCM patients improved functional capacity. [103] Neither study had serious events such as death or sustained VA and though they were not powered for safety, the RESET-HCM trial had three patients withdrawn due to NSVT.

Dejgaard et al. demonstrated that vigorous activity was associated with larger LV volumes, favorable diastolic function, and no increase in VA among 132 individuals with HCM, of whom 11 were competitive athletes. [104]

### What research has been done on HCM and exercise in athletes specifically?

Whereas most data in HCM come from sedentary individuals, several studies have evaluated HCM in athletes. In an observational study, Sheikh et al. demonstrated that competitive athletes with HCM had lower mean LV wall thickness, larger LV end-diastolic diameter and volume, more normal indices of diastolic function, lower LVOT gradients, less mitral regurgitation, a lower incidence of SAM, and similar proportion with LGE when compared to non-athletes with HCM [23]. Like the Dejgaard study mentioned previously, it is unclear whether these are a result of training or indicators of less severe disease which allow for high-level athletic training. A recent study described an effective high-intensity exercise program that favorably affected LV end-diastolic filling, stroke volume, and fitness in healthy, sedentary, middle-aged individuals; this suggests that improvements in diastolic function and preventing cardiac stiffness attributed to aging may mediate favorable cardiac adaptations in athletes with HCM [89, 105].

More recently, Pelliccia et al. showed that among 35 mostly low-risk athletes with HCM, there was no difference in the incidence of symptoms or major events between athletes who stopped exercise and those who continued competitive sports [106•]. This group published a follow-up study of predominantly low risk, white athletes demonstrating no difference in symptoms or freedom from SCA/D between HCM-trained and detrained patients. They noted that similar results may not be seen in populations that are younger, racially diverse, or have more severe phenotypes [107].

Data from the ICD Sports Registry showed that among 440 athletes with ICDs, there were no deaths or external resuscitations during or after sports and the likelihood of receiving a shock was similar between competition and other physical activity among individuals with HCM [84].

More data is needed to appropriately comment on the efficacy and safety of high-intensity exercise in this population. The Dallas HIIT-HCM Pilot Study (High Intensity Exercise for Increasing Fitness in Patients with Hypertrophic Cardiomyopathy; NCT03335332) will strive to address this gap in knowledge [89].

## Exercise and sporting guidelines

As more data emerge regarding the safety of exercise for individuals with HCM, American and European guidelines have evolved. Initial guidelines from the AHA/ACC in 2005 excluded athletes with “probable or unequivocal” HCM from all except low-intensity, “1A” sports, irrespective of risk modifiers or treatment [10]. European guidelines at the time forbade competitive sports in athletes with definite HCM but allowed athletes with a low-risk profile—no sudden death in relatives, no symptoms, mild LVH, normal blood pressure response to exercise, and no VA—to participate in low dynamic, low static, “1A” sports [8]. The American guidelines allowed genotype-positive–phenotype-negative individuals to participate in competitive sports, especially in the absence of cardiac symptoms or a family history of sudden death, whereas the European guidelines only allowed recreational, non-competitive sports for this population [8, 10].

The 2011 AHA/ACC HCM guidelines provided similar recommendations though also noted “it is reasonable for patients with HCM to participate in a range of recreation sporting activities” (Class IIa, Level of Evidence [LOE] C) [11]. The 2014 ESC-HCM guidelines still excluded athletes with HCM from competitive sports, though noted they “should maintain a healthy lifestyle” and that “advice on recreational activities should be tailored to symptoms and the risk of disease-related complications including SCA.” [1].

Updated recommendations were published by the AHA/ACC in 2015 and 2020 and by the ESC in 2020 [2••, 53••, 97]. These recommendations are detailed in Table 1.

**Table 1:** Evolution of AHA/ACC and ESC sport participation guidelines with HCM

Guideline	AHA/ACC Task Force 3 (2015)	ESC Sports Cardiology (2020)	AHA/ACC HCM (2020)
Genotype (+)/phenotype (−)	Participation in competitive sports is reasonable if asymptomatic and no family history of HCM-related SCD (IIa, C)	Participation allowed in all competitive sports (IIb, C)	Participation in competitive sports of any intensity is reasonable (IIa, C-LD)
Phenotype (+) High-intensity^#^ competition/exercise Low-moderate^#^ intensity competition/exercise	Participation not allowed—independent of age/sex/magnitude of LV hypertrophy/specific sarcomere mutation, presence/absence of LVOT obstruction, absence of prior symptoms, presence of LGE, history of septal reduction therapy (III, C)	Any markers of increased risk**: participation not recommended (II, C) No markers of increased risk**: after expert assessment, participation may be considered (except those where syncope may be associated with harm or death) (IIb, C)	High-intensity recreation/moderate-high-intensity competition: may be considered after comprehensive evaluation and shared discussion, repeated annually with an expert provider who conveys that the risk of sudden death and ICD shocks may be increased, and with the understanding that eligibility decisions for competitive sports often involve third parties (IIb, C-LD)
	Low-intensity (1A) sports allowed—independent of risk factors identified above (III, C)	Any markers of increased risk**: low-moderate-intensity recreation may be considered following expert assessment (IIb, C)	Mild-moderate-intensity recreation is beneficial (I, B-NR) Most patients with HCM: low-intensity competition is reasonable (IIa, C-EO)
Medications/ICDs for sole purpose of participation	Medications (i.e.: BB): should not be administered for sole purpose of facilitating participation in high-intensity sports and may interfere with maximum physical performance (III, C) Prophylactic ICDs for sole purpose of participation: not permitted. ICD indications for athletes with HCM should not differ from those in non-athletes with HCM (III, B)	Not specifically addressed	Prophylactic ICDs for sole purpose of participation in competitive athletics: should not be performed (III, B-NR)
Evaluation and follow-up	Not specifically addressed	Annual follow-up for individuals who exercise on a regular basis (I, C) and considered for genotype (+)/phenotype (−) individuals for phenotypic review and risk stratification Six-month follow-up for adolescents and young adults who are more vulnerable to exercise-related SCD (IIa C)	Comprehensive evaluation and shared discussion of potential risks of sports participation by an expert provider is recommended on an annual basis (I, C-EO)

*AHA*, American Heart Association; *ACC*, American College of Cardiology; *ESC*, European Society of Cardiology; *HCM*, hypertrophic cardiomyopathy; *SCD*, sudden cardiac death; *LD*, limited data; *EO*, expert opinion; *LV*, left ventricle; *LVOT*, left ventricular outflow tract; *LGE*, late gadolinium enhancement; *ICD*, implantable cardioverter defibrillator; *NR*, non-randomized; *BB*, beta blocker

*Guideline recommendations in parentheses (Class, Level of Evidence)

**Markers of increased risk: (1) Cardiac symptoms or history of cardiac arrest or unexplained syncope; (2) moderate ESC risk score (≥ 4%) at 5 years; (3) LVOT gradient at rest > 30 mmHg; (4) abnormal blood pressure response to exercise; (5) exercise-induced arrhythmias

^#^Examples of intensity: Low: bowling, golf; moderate: figure skating, rugby, running; high: kayaking, cycling, triathlon. See Levine et al. Task Force 1 Classification of Sport. *JACC* 2015 Dec 1;66(21):2350–2355 for full figure

## Shared decision-making

Individuals with HCM and their physicians must make decisions together regarding exercise, eligibility, SCA risk, and ICD implantation in the context of evolving data. As such, recent guidelines have shifted tone from generalized disqualifications to a nuanced focus on shared decision-making between patients, physicians, and relevant third parties to individualize decisions [2••, 53••]. The 2020 AHA/ACC HCM guidelines specifically encourage this to determine “participation in high-intensity recreational activities or moderate- to high-intensity competitive sports activities,” whereas prior recommendations categorically excluded them [2••]. Physicians should explain that risk varies based on age, race, specific sport, and phenotypic details and that existing risk calculators, such as the ESC-HCM SCA risk calculator, are derived from non-athletic cohorts and may not account for the specific stresses of exercise and high-intensity competition [1, 51, 53••]. Although recent guidelines allow for more liberalized participation, physicians should reiterate that SCA is possible even in the absence of all major risk factors [108]. Moreover, shared decision-making has limits; for example, patient autonomy is important but not without limitation especially considering that many young athletes may underestimate their individual vulnerability when faced with opportunities for fame and economic gain [56]. Other limits include the potential for disagreement among the athlete, team physicians and cardiologists, school or club and the delicate balance and legal ramifications regarding who has the final authority to permit or disqualify participation [56]. Ultimately, it is important that these decisions be made in concert with physicians experienced in both the cardiovascular care of athletes and patients with HCM and that all parties involved agree upon the final recommendation.

## Areas of further interest

While most data come from cohorts of sedentary individuals with HCM, future studies of athletes with HCM will provide relevant data for future recommendations. Moreover, the ultimate introduction of newer medical therapies such as mavacamten into local formularies will present an opportunity to evaluate the degree to which regression of hypertrophy changes the risk of SCA and other cardiovascular outcomes. Mavacamten inhibits cardiac myosin ATPase which reduces actin-myosin cross-bridge formation and reduces contractility. As a result, the EXPLORER-HCM trial showed it reduces LVOT gradients, improves symptoms, NYHA class, and peak VO2 in patients with obstructive HCM compared to placebo at 30 weeks [109, 110]. Notably, this trial was not done in athletes specifically. The mean age of participants was 58.5 years, likely much older than most athletes and > 90% in the mavacamten arm were white, which is not representative of athletes. It is unknown whether or how reverse remodeling will affect SCA risk.

Building upon existing ICD data to further characterize outcomes in HCM-specific populations will help inform treatment in this evolving area of debate. Finally, as the AHA/ACC and ESC have liberalized exclusions for moderate- to high-intensity exercise, further characterizing the safety of these activities for individuals with HCM will inform more data-driven recommendations.

## Conclusion

Differentiating HCM and EICR is challenging and often requires a nuanced interpretation of several diagnostic tools. Individuals with HCM may have an increased risk for cardiac arrest and identification of established risk factors is paramount for patient discussions and decisions. Correctly distinguishing pathology from expected remodeling has implications for activity recommendations and treatment. Whereas previous guidelines from the AHA/ACC and ESC have excluded individuals with HCM from moderate- to high-intensity sports and competition, recent data and a focus on shared decision-making have led to the most recent guidelines offering and individualized approach for this population as a whole and among individuals with ICDs. Further studies of athletes with HCM and ICDs as well as the risk reduction profile of emerging therapies such as mavacamten will meaningfully improve the ability to risk stratify and appropriately manage athletes with HCM.
